# Somatostatin Improved B Cells Mature in Macaques during Intestinal Ischemia-Reperfusion

**DOI:** 10.1371/journal.pone.0133692

**Published:** 2015-07-29

**Authors:** Ling Liu, Qinghua Tan, Bin Hu, Hao Wu, Chunhui Wang, Rui Liu, Chengwei Tang

**Affiliations:** 1 Department of Gastroenterology, West China Hospital, Sichuan University, Chengdu, China; 2 Division of Peptides Related with Human Diseases, Key Laboratory of Biotherapy of Human Diseases, Ministry of Education, West China Hospital, Sichuan University, Chengdu, China; University of Cordoba, SPAIN

## Abstract

**Aims:**

Intestinal ischemia-reperfusion has been taken as an important pathophysiological process for multiple organ dysfunctions in critical patients. Recent studies reported that dual expression programs of the B cells receptors and Toll-like receptors on B-lymphocytes permit these ubiquitous cells to integrate both adaptive and innate immune functions. Our previous studies found that somatostatin inhibited the intestinal inflammatory injury after ischemia-reperfusion in macaques. However, the changes of B cells and the effects of somatostatin on B cells after intestinal ischemia-reperfusion were unclear.

**Methods:**

15 macaques were divided into control, intestinal ischemia-reperfusion and somatostatin pretreatment groups. Immunohistochemistry was performed to identify the distributions of adaptive and innate immunity markers in the iliac mucosa. Hmy2.cir B lymphoblastoid cell line was cultured *in vitro* study. Enzyme-linked immunosorbent assay was used to measure IgM, IL-6 and SIgA, and the expressions of B cells transcription factors, PAX-5 and BLIMP-1, were detected by Western blotting.

**Results:**

B2 lymphocytes in normal Peyer’s patches were presented the phenotype of PAX-5^+^CD20^+^CD5^-^. Ischemia-reperfusion increased the numbers and sizes of Peyer’s patches but with PAX-5^+^CD20^-^CD5^-^ B cells, an unmatured set of B cells. Somatostatin partly kept the phenotype of mature B cells during ischemia-reperfusion. The innate immunity of B cells was inhibited whereas the adaptive immunity was increased in the intestinal mucosa in the somatostatin group, compared to the ischemia-reperfusion group. *In vitro*, somatostatin significantly inhibited IL-6 and promoted IgM by increasing the expression of both PAX-5 and BLIMP-1 in the proinflammatory condition.

**Conclusion:**

Intestinal ischemia-reperfusion resulted in the proliferation of unmatured B cells which were involved in the augmentation of innate immunity. Somatostatin, with a bi-directional regulation function on innate as well as adaptive immunity of B cells, greatly improved B cells mature in macaques during ischemia-reperfusion. Preventive supplements of somatostatin may greatly limit intestinal injury and bacterial translocation during ischemia-reperfusion.

## Introduction

Intestinal ischemia-reperfusion (IIR) is an important pathophysiological process in clinically critical illness, including severe trauma, shock, severe acute pancreatitis, infection, organ transplantation and other inflammatory-related diseases[1]. Over the past two decades, clinical and basic studies have identified the gut as the "motor" of systemic inflammatory response syndrome (SIRS) and multiple organ dysfunction syndromes (MODS) in these critical conditions [2–4]. It has been reported by our group and other groups, that exaggerated intestinal innate immune responses involving Toll-like receptor 4(TLR4) and proinflammatory cytokines contribute to massive intestinal and systemic inflammatory injury [5–8]. However, little is known regarding the role of adaptive immunity after IIR.

B cells are ubiquitously identified as key participants in the adaptive immune response. Traditionally, it has been widely accepted that the role of B cells in gut adaptive immune function is to secrete antibodies, particularly from the IgA class, that inhibit the colonization of pathogenic microorganisms and the penetration of harmful luminal antigens, such as bacterial toxins. B2 cells are the most prominent B cells in the peripheral lymphoid organs [9, 10]. These cells express B cell receptors (BCRs) and primarily participate in T-dependent responses, leading to the generation of high-affinity antibodies and long-term memory. Recent studies have also reported that some B cells, including B1 cells in peritoneal and pleural cavities, bone marrow perisinusoidal, and splenic marginal zones and B cells in patients with autoimmune diseases, highly express TLRs, which produce polyreactive IgM against conserved sequence antigens in a T cell-independent manner[11–14]. Additionally, studies have reported that B cells expressing TLRs produce regulatory cytokines and/or chemokines, such as interleukin (IL)- 6, transforming growth factor-β, CXCR4 and IL-10 [15–19]. Therefore, the dual expression programs of BCRs and TLRs, permit B cells to uniquely integrate both adaptive and innate immune functions. Previous studies have been suggested that IIR injury is initiated by recognition of natural IgM and B1 cells are a major source of pathogenic IgM [20, 21]. Notably, reconstitution of IgM or an enriched fraction of B1 cells restores injury [20, 21]. However, as basic and clinical research has evolved, the role of B cells in the mechanism of intestinal and systemic inflammatory injury after IIR remains unclear.

Somatostatin (SST), a 14-amino acid polypeptide, is now known to be a multifunctional neuropeptide. We used both IIR rat and macaques models to confirm that SST functions to prevent innate inflammatory injury in the intestinal mucosa [7, 8]. Furthermore, we found that SST concentrations in the intestine were positively correlated with the maturity of the macaques’ Peyer’s patches (PPs), which are generally considered to represent inductive sites for intestinal immune responses[22, 23]. Previous research has also shown that SST strongly bound the cell populations in PPs [24] and inhibited the proliferation of lymphocytes and IgM synthesis of the cells in PPs [25]. Because B lymphocytes have been shown to predominate in the follicular germinal centers of PPs[26] and SST-receptors have been found in B lymphoblast cells[27, 28], we were intrigued by the idea that SST is one of the most crucial microenvironmental factors for modulating the B cell function in the gut.

In this study, we observed the effects of SST on B cells in the gut in an IIR model *in vivo*. Additionally, the function of SST on B cells function was studied in *vitro*. Using both *in vivo* and *in vitro* data, we discovered that SST is an important regulator in both the adaptive and innate immunity of B cells. To provide greater clinical relevance, a macaque IIR animal model was used in this study.

## Materials and Methods

### Ethics statement

Healthy adult rhesus macaques (4–7 years, body weight 6.9±1.7 kg, male/female = 9/6) were obtained from the Animal Center of Sichuan University. The experiments in this study were performed in accordance with the guidelines of the Sichuan University Institutional Animal Care and Use Committee (IACUC) and all experiments were received a permit from the Sichuan University IACUC. All animals were housed in an environment with a temperature of 20–22°C with 12 h light/dark cycles in same pairs to allow for social interactions. Cages met certain spatial requirement and ensured a certain amount of diversion, freedom of movement and safety (length × width × height = 1 × 1.8 × 1 m). All animals were fed twice a day (early morning and noon) with commercial monkey chow supplemented with fruits. Toys and branches were provided in indoor cages. The animals were anesthetized with xylazine (0.2 ml/kg, i.m.) and maintained with diazepam (0.1 ml/kg, i.v.) and carbrital (30 mg/kg, i.v.) for 24 hours, as needed to alleviate suffering. Veterinary monitoring included responsibility for maintenance of appropriate health records, provision of advice on anesthesia regimes, and assistance with technical and surgical procedures during the entire experiment. Because all procedures and euthanasia were performed completely under anesthesia, death is used as the clinical endpoint in our experiment. The animals were sacrificed 24 hours after IIR by an overdose of anesthesia, and the specimens were later removed.

### IIR Surgical Procedures in Macaques

As previously described in detail [8], a midline laparotomy of 5 cm in length was performed. Then, the superior mesenteric artery (SMA) was isolated and occluded with a microsurgical clip. After occlusion for 1 hour, the clip was removed, and intestinal perfusion was reestablished. A catheter was placed in a peripheral vein to infuse 0.9% saline and 20 g glucose (0.1∼0.2 ml/kg/min, i.v. gtt) for 24 hours during the process.

### Experimental Grouping

As previously described in detail [8], fifteen macaques were randomly divided into three groups, with five animals (male/female = 3/2) in each group. In the normal control (NC) group, the animals underwent a sham operation with the same treatment described above, except that the IIR procedure was not performed. In the IIR group, the animals underwent the IIR procedure. In the IIR+SST group, SST-14 (Serono Singapore Pte Ltd, Singapore) was intravenously administered to the animals at a dosage of 5 μg/kg/h from 5 min before SMA occlusion until the end of the experiment.

### Plasma endotoxin assay

Blood samples from the portal veins were collected in heparin-containing tubes, and plasma was separated by centrifugation at 2500 rpm for 10 min. Plasma endotoxin concentrations were measured using a commercially available quantitative chromogenic endpoint Limulus Amebocyte Lysate kit (Yihua medical technology company, Shanghai, China). Briefly, 100μl plasma was diluted with 200ul endotoxin free water and 200ul Tris-HCL buffer, then heat inactivated at 100°C for 10 min. The supernatant was separated by centrifugation at 3000rpm for 10 min. Remaining procedures were performed according to the manufacturer’s instructions. Endotoxin concentrations (EU/ml) in the samples were determined from a standard curve using pure endotoxin standards by spectrophotometer (Type 721) at 545nm.

### HE staining of Peyer’s patches (PPs) in the ileum

The terminal ileum of each animal was removed and fixed in 10% formaldehyde. The histological sections were evaluated in a blinded fashion. For the quantitative evaluation of size and numerical changes in the terminal ileum, the PPs in each animal were quantified with Image-Pro Plus 4.0 software.

### Visualization of paired box protein(PAX)-5, CD20, CD5, plasma cells, IgA, secretory components, TLR4, NF-κB(nuclear factor-κ-gene binding) and interferon(IFN)-γ by immunohistochemistry

As previously described in detail [8], sections of the terminal ileum were deparaffinized and microwaved for 15 min. For nonspecific blocking, 10% goat serum was added, and the sections were incubated for 20 min at room temperature. The following antibodies were then added to the individual sections: mouse anti-human PAX-5, CD20, CD5, plasma cells (monoclonal antibody, 1:100, Zymed, California, USA), goat anti-rhesus IgA2 (Nordic Immunological Laboratories, Tilburg, Netherlands), goat anti-rhesus secretory components (Nordic Immunological Laboratories, Tilburg, Netherlands), rabbit anti-human TLR4 and NF-κB-p65 (polyclonal antibody, 1:100, Zymed, California, USA), and rabbit anti-human IFN-γ (polyclonal antibody, 1:200, Boster Bioagent Company, Beijing, China). After incubation with these antibodies for 120 min at 37°C and overnight at 4°C, the sections were stained with a ready-to-use streptavidin-catalase immunohistochemical reagent system. The color reactions were developed with diaminobenzidine (DAB; Zhongshan Bioagent Company, Beijing, China).

The PAX-5 positive cells or CD20 positive cells were the cells appearing as brown granules on the membrane or nucleus, respectively. The TLR4 positive cells were the cells appearing as brown granules on the membrane and cytoplasm and the NF-κB in the cytoplasm and the nucleus. The IFN-γ positive cells were the cells appearing as brown granules on the cytoplasm. A semi-quantitative immunohistochemical analysis for all of the PPs in the terminal ileum was performed with Image-Pro Plus 4.0 software. The integrated optical density (IOD) values of markers for cells, IgA, secretory components, PAX-5, CD20, CD5, TLR4, NF-κB and IFN-γ were scored. Each value represented the mean IOD ± SD of five visual fields in one macaque, from which duplicate measurements were performed. For the analysis of PAX-5, CD20, CD5 TLR4, NF-κB and IFN-γ markers, PPs were selected at low magnification (×100) and the positive-staining cells were calculated at high magnification (×200).

### Cell culture and in vitro study

Hmy2.cir, a human B lymphoblastoid cell line in the late stage of B cell development, was derived from the HMy.2 B-LCL by γ-irradiation and selected for class I loss by antibody and complement. The cell line expresses no HLA-A or-B locus gene products[29]. It was cultured in IMDM (Iscove's Modified Dulbecco's Medium) containing 10% fetal calf serum, 4 mM L-glutamine, 1.5 g/L sodium bicarbonate and penicillin (100 U/ml)/streptomycin (100 μg/ml) in a humidified 5% CO2 atmosphere at 37°C. Hmy2.cir cells were incubated with the stimulation of lipopolysaccharides (LPS) (Sigma-Aldrich, Minnesota, USA), SST (Laboratoires Serono, Geneva, Switzerland), and IFN-γ (Roche, Basle, Switzerland). Experiments were divided into 3 pairs: control *vs* SST (10^−9^ mol/L); LPS (10 μg/ml) *vs* LPS (10 μg/ml)+SST (10^-9^mol/L); LPS (10 μg/ml)+IFN-γ (500 μ/ml) *vs* LPS (10 μg/ml)+IFN-γ (500 μ/ml)+SST (10^-9^mol/L).

A total of 1x10^5^ cells were incubated in 96-well plates for 72 hours, and each sample was refreshed every day and tested in triplicate. The standard MTT (3–2,5 diphenyl tetrazolium bromide) l (Amresco, Ohio, USA) method was used to detect cell viability at the concentration of 5 mg/ml. The absorbance in each group was read at 590 nm with a reference filter of 620 nm. The viability index was calculated as the ratio of the absorbance in the experimental group to that in the blank control group.

Next, 3 ml of 2x10^5^ cell suspension was plated into each well in a 6-well flat-bottomed plate under previously described conditions,and each sample was refreshed every day and tested in triplicate. After incubation and stimulation for 72 h, the cell culture supernatants were collected; the particles were removed by centrifugation; and the samples were stored at -80°C.

### Determination of IgM, IL-6 and secretory IgA (SIgA) in the supernatant by Enzyme-Linked Immunosorbent Assay (ELISA)

Expression levels of IgM, IL-6 in cell culture supernatant and SIgA in the mucosa scraped from the terminal ileum were determined with an ELISA kit (R&D Systems, Inc., Minneapolis, USA), according to the manufacturer’s instructions.

### Expression of BLIMP-1, PAX-5 in the B cells by Western blotting

Frozen cell pellets from the *in vitro* study were sonicated in buffer (50 mM Tris-HCl) containing 150 mM NaCl, 1 mM Na_3_VO_4_, 1 mM PMSF and a protease inhibitor cocktail (Sigma-Aldrich, Minnesota, USA) before being centrifuged at 3000×g for 10 min. Supernatants were collected as total mucosal proteins, and the protein concentrations were measured using Bio-Rad Protein Assays. The proteins were separated on 10% SDS polyacrylamide gels using a Bio-Rad system apparatus and blotted onto nitrocellulose membranes. Nitrocellulose membranes were incubated in TBST-milk (TBS buffer containing 0.1% Tween-20 and 5% milk), followed by primary antibodies, goat anti-human BlIMP-1 polyclonal antibody (1:250, Abcam, Cambridge, UK), and rabbit anti-human PAX-5 monoclonal antibody (1:100, Labvision, California, USA). Blots were then washed with TBST 3 times (15 min each) and subsequently incubated (1 hour) with corresponding horseradish peroxidase-labeled secondary antibodies (Zymed, California, USA), which were diluted in TBST. The blots were washed 4 times (10 min). Using Western blotting luminal reagent (Santa Cruz, Texas, USA), the chemiluminescent intensity of the detected protein bands was detected by a film developer.

### Statistical Analysis

All the quantitative data are presented as the means ± SD from the five animals in each group. The data were analyzed using SPSS (version 14.0; SPSS, Inc., Cary, NC, USA). The data were evaluated with one-way analysis of variance (ANOVA) and confirmed using Tukey’s test for multiple comparisons. Significance was set at *p*<0.05.

## Results

### Somatostatin ameliorated the inefficient proliferation of iliac PPs during IIR

**Fig 1:**
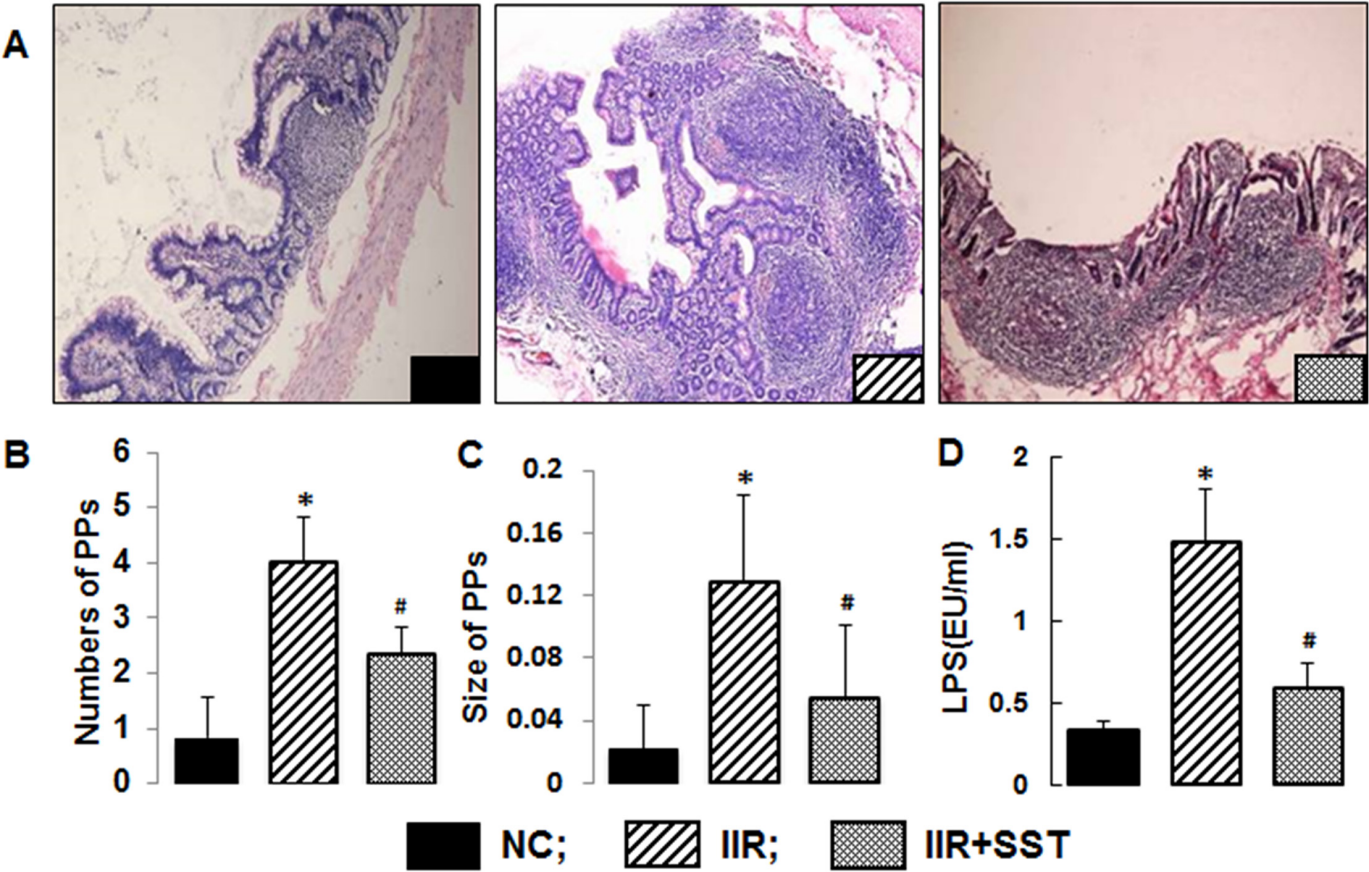
The effects of IIR and IIR+SST on the changes of PPs and LPS level in portal vein. A) Visualization of PPs in the three groups (HE staining, ×100 magnification); B) Quantitative comparison of ileac PPs numbers in the three groups; C) Quantitative comparison of ileac PPs size in the three groups; D) LPS levels of portal veins in three groups. Data represent the mean ± SD of 5 animals per group. *, *vs NC* group, *P*<0.05; ^#^, *vs IIR* group, *P*<0.05.

Compared to the NC group, the numbers and sizes of iliac PPs in the IIR group were significantly increased, *p*<0.05. Those changes in IIR group were greatly reduced in the IIR +SST group, *p*<0.05 (Fig 1). Interestingly, the LPS level of portal vein in IIR group was highest among three groups (*p*<0.05). SST treatment significantly decreased the LPS level of portal vein affected by IIR (*p*<0.05) and made that no difference with NC group (*p*>0.05) (Fig 1).

### Somatostatin partly kept the phenotypes of mature B cells during IIR

**Fig 2:**
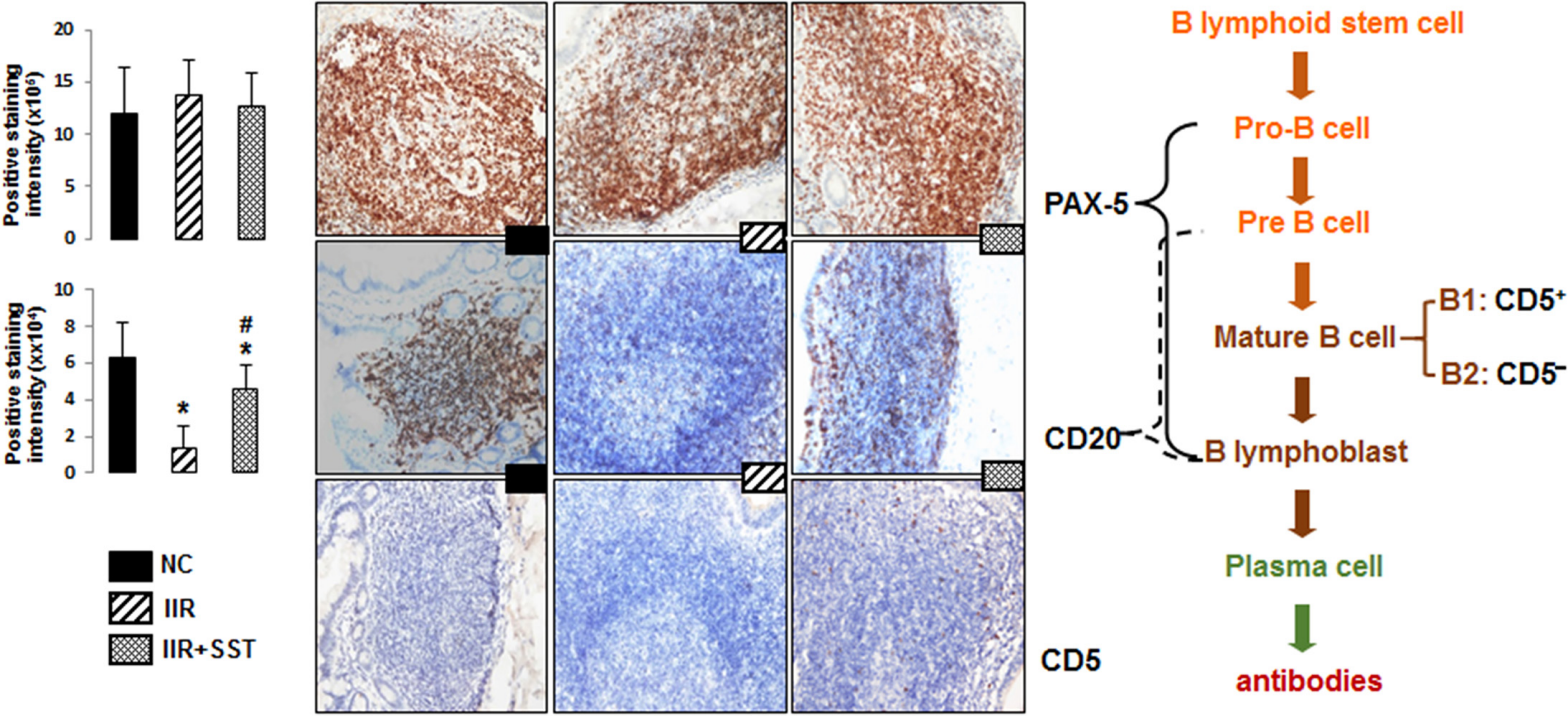
The changes of phenotypes of B cells in three groups. Expressions of PAX-5, CD20 and CD5 in PPs of the terminal ileum were greatly changed during *IIR* when compared with normal control (*NC*) group. SST intervention partly kept the phenotypes of mature B cells. (Immunohistochemical staining, ×200 magnification). Positive staining intensity in the PPs were represented as the mean ± SD of 5 animals per group. *, *vs NC* group, *P*<0.05; ^#^, *vs IIR* group, *P*<0.05.

The terminal ileum was stained with antibodies for PAX-5, CD20 and CD5 in sections to identify the characteristics of the cells in the proliferated PPs. B cells in NC group presented the phenotype of PAX-5^+^CD20^+^CD5^-^, which were considered as B2 lymphocytes (Fig 2). Although PPs were greatly proliferated during IIR, the phenotypes of B cells within the germinal center of PPs were different from those of normal control group. The CD20^+^ staining cells in the PPs after IIR were significantly decreased compared to the NC group,*p*<0.05,whereas no significant difference of the PAX-5^+^ staining cells was found between the two groups. IIR attack stimulated the proliferation of PAX-5^+^CD20^-^CD5^-^ cells, an unmatured set of B cells in the PPs. SST treatment partly improved B cells mature with increased of CD20^+^ staining in the PPs during IIR, *p*<0.05, (Fig 2).

### Somatostatin was helpful to preserved humoral immune response of B cells during IIR

**Fig 3:**
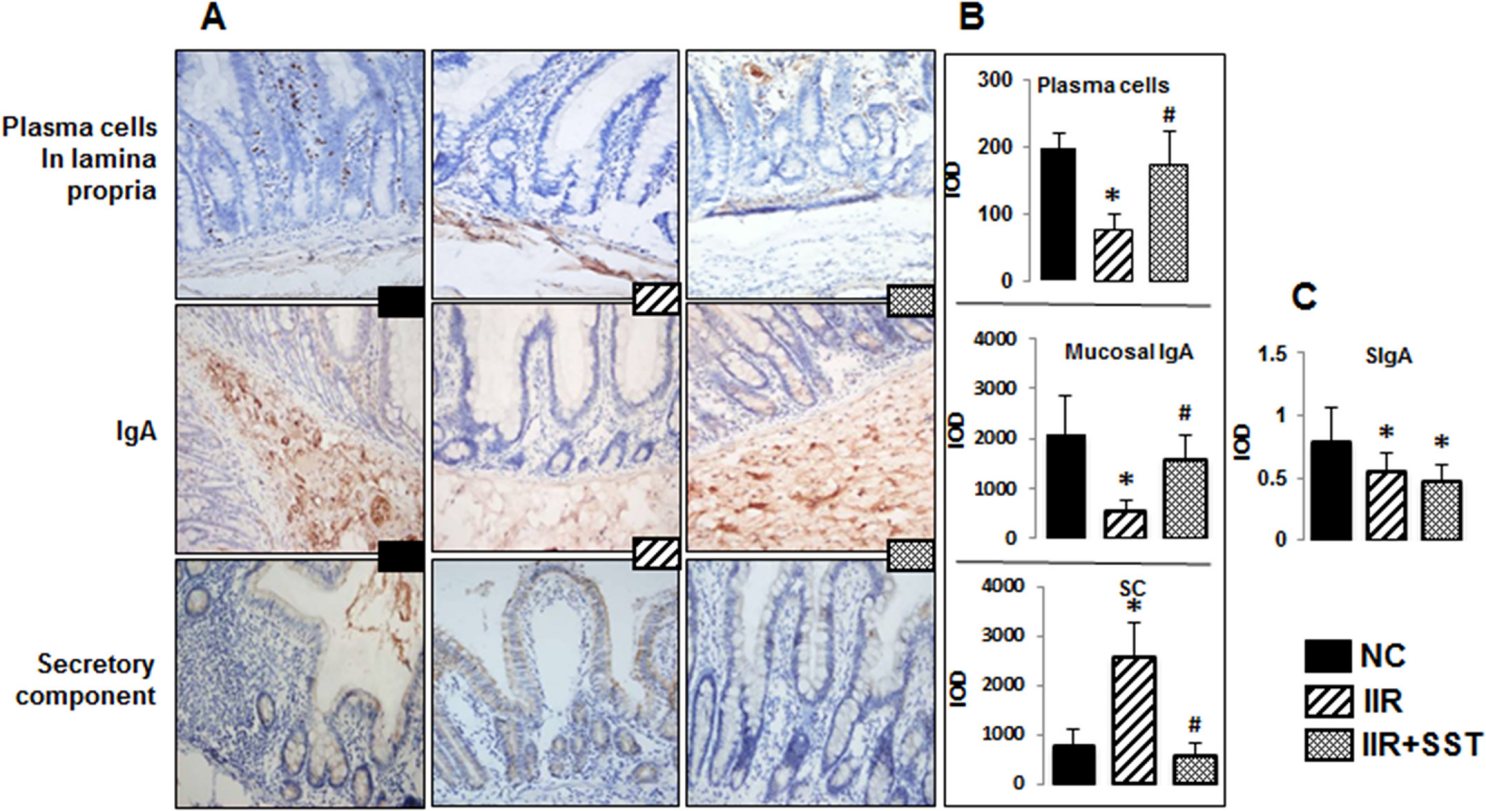
Humoral immune response of B cells in three groups. A) Location of plasma cells, IgA and secretory components in the terminal ileum of three groups. (Immunohistochemical staining, × 200 magnification). B) Semi-quantitative analysis of the plasma cells in the lamina propria, IgA in the submucosal area and secretory component (SC) in the intestinal lumen by integrated optical density (IOD) in the three groups. C) SIgA levels in the ileac mucosa measured by ELISA. Data were showed as the mean ± SD of 5 animals per group. *, compared with the *NC* group, *P*<0.05. ^#^, compared with the *IIR* group, *P*<0.05.

Immunohistochemical staining revealed that intestinal plasma cells of the NC group were located mainly in the lamina propria; IgA was located in the submucosal area and the secretory components were located in the intestinal lumen (Fig 3A). During IIR, fewer plasma cells moved from submucosa to lamina propria. Quantitation of IgA or SIgA in IIR group were significantly decreased compared to the NC group, *p*<0.05 (Fig 3A and 3B and 3C). The plasma cells and the secretion of IgA in the IIR+SST group were increased in the intestinal mucosa, compared to the IIR group, *p*<0.05 (Fig 3B). Due to the significantly decreased level of the secretory components in the IIR+SST group compared to the IIR group, SIgA was not recovered to the normal level in the IIR+SST group (Fig 3B and 3C).

### Somatostatin inhibited the activated innate immunity of B cells during IIR

**Fig 4:**
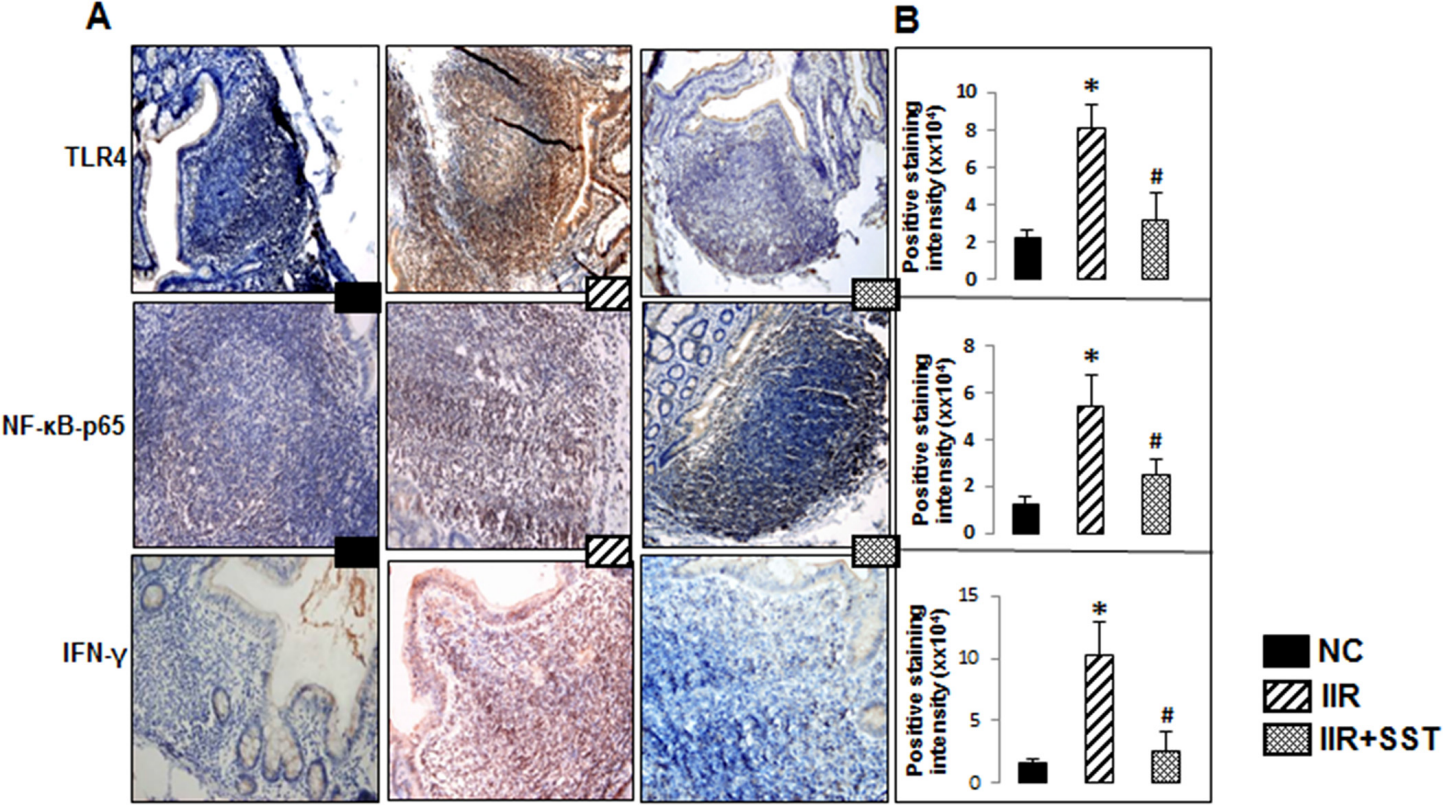
The innate immune response in PPs in three groups. A) Expressions of TLR4, NF-κB and IFN-γ in the PPs of the terminal ileum in three groups (Immunohistochemical staining, ×200 magnification). B) Semi-quantitation for positive staining intensity of TLR4, NF-κB and IFN-γ in the PPs. Data were showed as the mean ± SD of 5 animals per group. *, compared with the *NC* group, *P*<0.05. ^#^, compared with the *IIR* group, *P*<0.05.

The expressions of TLR4, NF-κB and IFN-γ in macaque intestine were significantly increased in the IIR group, compared to their weak expressions in the NC group, *p*<0.05 (Fig 4A and 4B). SST treatment significantly down-regulated the expressions of TLR4, NF-κB and IFN-γ in the PPs, compared to the IIR group, *p*<0.05 (Fig 4A and 4B). Therefore, with the supplementation of SST after IIR, these data support that, the activated innate immunity in the germline centers of PPs was significantly inhibited.

### Somatostatin promoted the development of plasma cells and production of IgM by suppression of IL-6 *in vitro*

**Fig 5:**
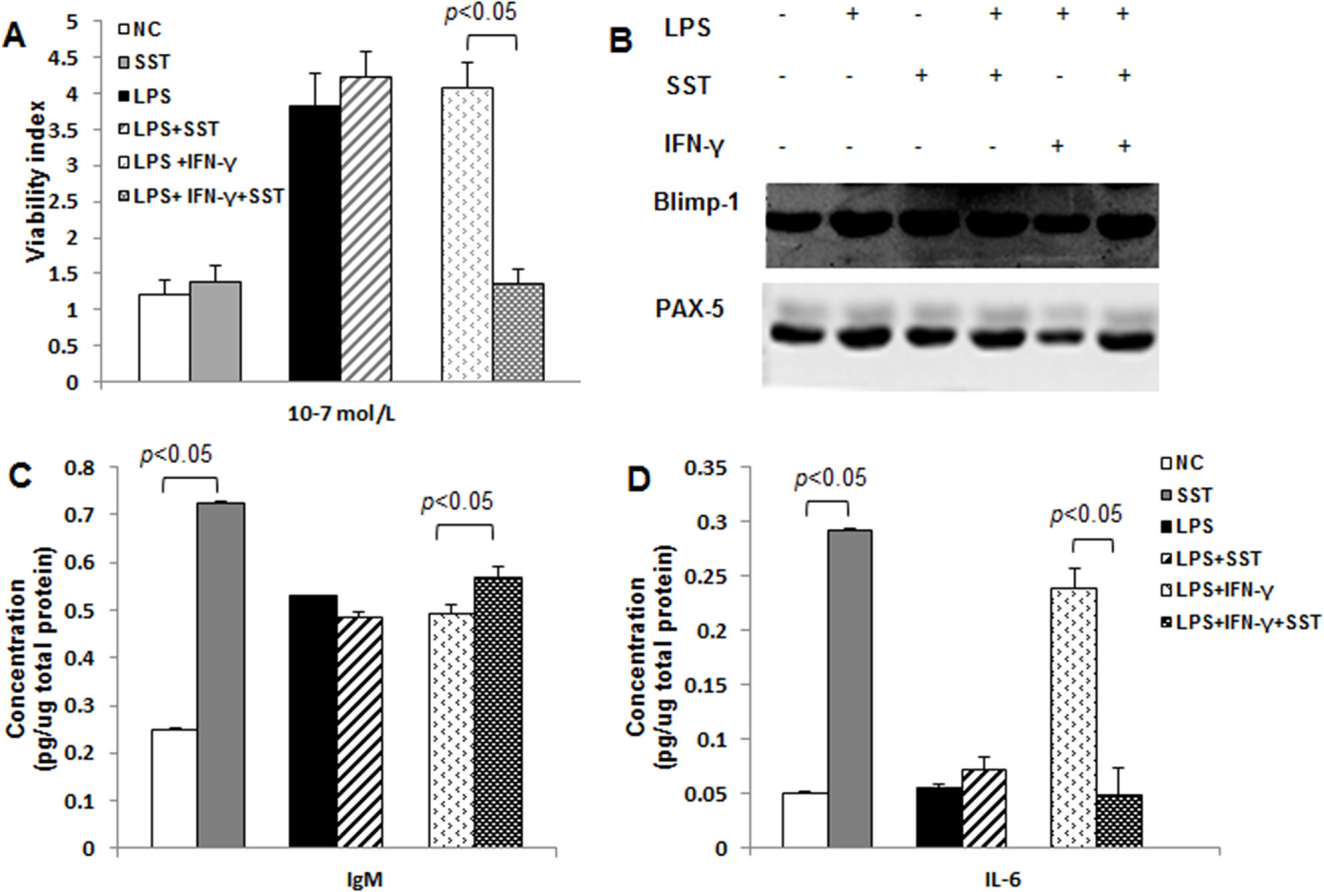
The effects of SST on the viability of Hmy2.cir, expression of PAX-5, Blimp-1, IL-6 and production of IgM *in vitro*. Hmy2.cir, a human B lymphoblastoid cell line, were incubated with LPS (10 μg/ml), SST (10^−9^ mol/L) and IFN-γ (500 μ/ml) for 72 h. Experiments were divided into three pairs of group: control *vs* SST; LPS *vs* LPS+SST; LPS+IFN-γ *vs* LPS+IFN-γ+SST. A) Viability *of* Hmy2.cir measured by MTT assay in three pairs of groups. B) Expression of PAX-5 and Blimp-1 in three pairs of groups (Western blotting). C) IgM concentration in three pairs of groups (ELISA). D) Expressions of IL-6 in three pairs of groups (ELISA). Representatives of three independent experiments were shown and the experiments were triplicated.

There was no effect of SST (10^−9^ mol/L) on the viability of Hmy2.cir, a human B lymphoblastoid cell line in the late stage of B cell development. Either LPS alone or combined with IFN-γ significantly increased the viability of Hmy2.cir, *p*<0.05. However, SST only dramatically reduced the viability of Hmy2.cir stimulated by LPS+IFN-γ (Fig 5A).

Similar effect of SST on the expression of PAX-5 or Blimp-1 was also observed in Hmy2.cir cell line. Differently, LPS alone greatly stimulated the expression of PAX-5 or Blimp-1(*p*<0.05), but LPS+IFN-γ significantly inhibited the expression of PAX-5 or Blimp-1(*p*<0.05), which could be efficiently prevented with the addition of SST, *p*<0.05 (Fig 5B).

The IgM concentration in SST was significantly higher than that in NC group, *p*<0.05. There was no significant difference of IgM concentration between LPS and LPS+SST, *p>*0.05. LPS+IFN-γ+SST greatly increased IgM production when compared with LPS+IFN-γ, *p*<0.05. In contrast to IgM, LPS+IFN-γ+SST dramatically decreased IL-6 production when compared with LPS+IFN-γ, *p*<0.05 (Fig 5C and 5D). The effects of SST on viability, IgM and IL-6 secretion in LPS+IFN-γ condition were consistent over different concentrations (10^−7^, 10^−9^, 10^−11^ mol/L) and time points (24, 48 and 72 h), as shown in supplementary data (S1 Fig).

## Discussion

The exaggerated intestinal innate immune responses after IIR have been described both extensively and intensively [2–4]. However, the adaptive humoral immune response during IIR is still less concerned. PPs constitute important structures of the gut-associated lymphoid tissue (GALT). Based on their anatomical location distributed along the intestinal tract and their contribution to IgA production, PPs form the first front of mucosal immunity and play an important role in gastrointestinal immune defense. Portal circulating endotoxins (LPS) is usually taken as a marker of bacterial translocation. In the macaque experiment, IIR attack greatly increased the numbers and sizes of PPs while did not resist invasion of LPS into portal vein, probably indicating an inefficient proliferation of iliac PPs. Interestingly, somatostatin significantly ameliorated such inefficient proliferation of iliac PPs.

Bone marrow is the principal location of postnatal B-cell development, encompassing the process from progenitor, pre-B cells, and immature B cells to mature B cells. Recently, it has been reported that early B-cell development also occurs within the mouse gut, where antigens from gut microbes are available for interactions, possessing a window of opportunity for shaping the receptor-editing process [30]. In the initial experiments of this study, sets of B cell markers were screened for expression on the surface of splenic follicular and marginal zone B cells in macaques as a positive control. Because of the stable, organ-specific expression of the B cells of macaques, four B cells markers, including PAX-5 (pro-B cells to B lymphoblast marker)[31], CD20 (pre-B cells to B lymphoblast marker), CD5 and plasma cells, were selected to identify the different stages of B cell development or activation. The dada from this study indicated that IIR stimulated the proliferation of unmatured B cells in PPs, which differentiate into plasma cells poorly. The unique antibody, secretory IgA (SIgA), is the hallmark of gut-associated lymphoid tissue immune responses. Because of the differentiation obstacle of B cell—plasma cells, SIgA, the most abundant antibody in mucosal secretions was also dramatically decreased during IIR. B cells could not exert their immune functions. The defective adaptive immunity in the intestinal mucosa may partly interpret the high level of LPS in portal circulation.

Although several studies have reported that B cells with TLRs expression could produce polyreactive IgM, regulatory cytokines and/or chemokines [11–16, 18, 32], they did not concern the relationship of B cell development and its function of innate immunity. Considering that the PAX-5^+^CD20^-^CD5^-^ cells were the major B cell phenotypes after IIR in the germinal centers of PPs of the terminal ileum, our data suggested that the unmatured B cells proliferated during IIR might be involved in the augmentation of innate immunity through the TLR4-NFκB-proinflammatory cytokines pathway. These results greatly convert our traditional understanding for B cell-mediated immune response.

SST, a multifunctional neuropeptide, is mainly released from the sensory nerve endings and gastrointestinal neuroendocrine cells. This study further indicated that SST greatly improved B cells mature and its immune function in the proliferated PPs during IIR. Moreover, SST seems worked as a switch between humoral adaptive immune and innate immune response in B cells. IIR attacks cause shortages of endogenous SST in the plasma and intestinal mucosa of macaques [7] and resulted in a massive innate immune response involved by B cells of PPs. Preventive supplements of SST may greatly limit intestinal injury and bacterial translocation not only by inhibition of massive innate immune response but also by enhancement of humoral immune function of B cells.

SST is an important local factor to modulate gut immunity [7, 8, 22, 24, 33]. Similar as *in vivo*, the experimental inflammation (LPS+IFN-γ) greatly stimulated the viability of B cells (Hmy2.cir) *in vitro*. SST only dramatically reduced the viability of Hmy2.cir stimulated by LPS+IFN-γ but not by LPS alone, indicating the media effect of IFN-γ. Also, IFN-γ suppressed the mature of B cells with the reduction of PAX-5 or Blimp-1 expression. Not only SST greatly improved B cells mature and its immune function in this *in vivo* study, but also efficiently prevented the reduction of PAX-5 or Blimp-1 expression *in vitro*.

As a common cause of systemic inflammatory response and natural immunity[34], LPS can directly stimulate the proliferation, differentiation and maturation of B cells in the absence of other cells and can secret IgM antibodies and cytokines *in vitro*[35, 36]. SST stimulated Hmy2.cir to produce IgM and proinflammatory cytokine IL-6. In contrast, SST reduced the production of IL-6 in the LPS+IFN-γ condition *in vitro*. Due to SST receptors have been found on B cells [27, 28], the functions of SST might be conducted directly or indirectly.

## Conclusion

IIR resulted in the proliferation of unmatured B cells which were involved in the augmentation of innate immunity through the TLR4-NFκB-proinflammatory cytokines pathway. SST, with a bi-directional regulation function on innate as well as adaptive immunity of B cells, greatly improved B cells mature in macaques during IIR. Preventive supplements of SST may greatly limit intestinal injury and bacterial translocation during IIR attack.

## Supporting Information

S1 Fig SST consistently inhibited the viability and innate immunity of B cells in the proinflammatory condition over different concentrations and time points. Hmy2.cir, a B lymphoblastoid cell line, were incubated with LPS (10 μg/ml), SST and IFN-γ (500 μ/ml). Experiments were divided into three pairs of group: control *vs* SST; LPS *vs* LPS+SST; LPS+IFN-γ *vs* LPS+IFN-γ+SST. A) Comparison of viability index by MTT assay over different SST concentrations (10^−7^ mol/L and 10^−11^ mol/L) for 72 h. B) Comparison of viability index by MTT assay over different time points (24 and 48 h) in 10^−9^ mol/L. C) Comparison of IgM and IL-6 in the supernatant over different concentration (10^−7^ mol/L and 10^−11^ mol/L). Representatives of three independent experiments were shown and the experiments were triplicated.
(TIF)
