# Supplementary figures and images for: Somatostatin Improved B Cells Mature in Macaques during Intestinal Ischemia-Reperfusion
Source: PLoS One. 2015 Jul 29;10(7):e0133692. doi: 10.1371/journal.pone.0133692 (PMC4519283; doi:10.1371/journal.pone.0133692)

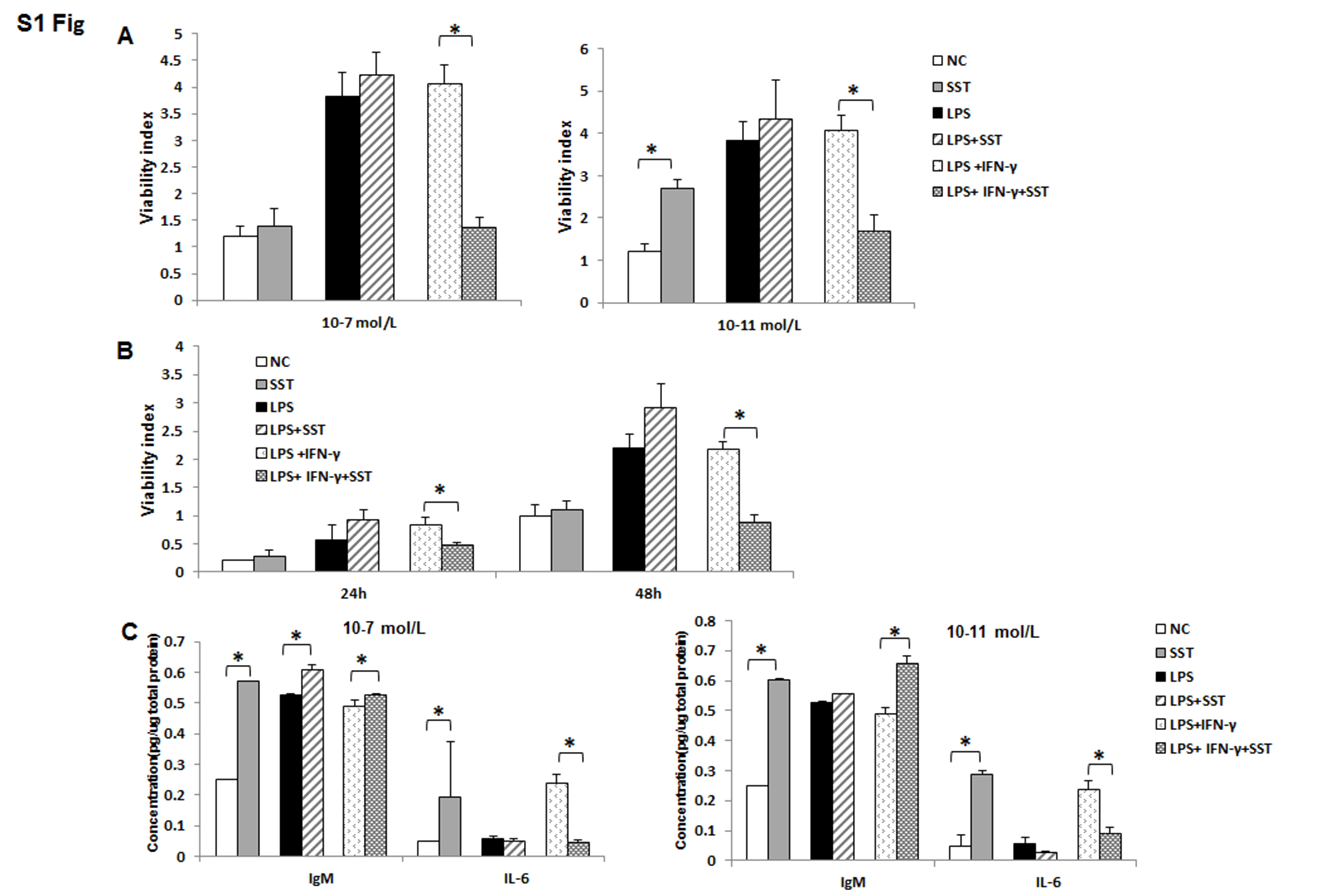

Supplement: S1 Fig — Hmy2.cir, a B lymphoblastoid cell line, were incubated with LPS (10 μg/ml), SST and IFN-γ (500 μ/ml). Experiments were divided into three pairs of group: control vs SST; LPS vs LPS+SST; LPS+IFN-γ vs LPS+IFN-γ+SST. A) Comparison of viability index by MTT assay over different SST concentrations (10−7 mol/L and 10−11 mol/L) for 72 h. B) Comparison of viability index by MTT assay over different time points (24 and 48 h) in 10−9 mol/L. C) Comparison of IgM and IL-6 in the supernatant over different concentration (10−7 mol/L and 10−11 mol/L). Representatives of three independent experiments were shown and the experiments were triplicated. (TIF) [file pone.0133692.s001.tif]
